# Supplementary material for: Transcription initiation profiling defines the regulatory logic of astrocyte gene regulation
Source: bioRxiv. 2026 May 28:2026.05.03.722406. Originally published 2026 May 4. Preprint. [Version 2] doi: 10.64898/2026.05.03.722406 (PMC13174308; doi:10.64898/2026.05.03.722406)
Supplement: Supplement 6 [file NIHPP2026.05.03.722406v2-supplement-6.pdf]

bioRxiv preprint doi: https://doi.org/10.64898/2026.05.03.722406; this version posted May 28, 2026. The copyright holder for this preprint (which was not certified by peer review) is the author/funder, who has granted bioRxiv a license to display the preprint in perpetuity. It is made available under a CC-BY 4.0 International license.

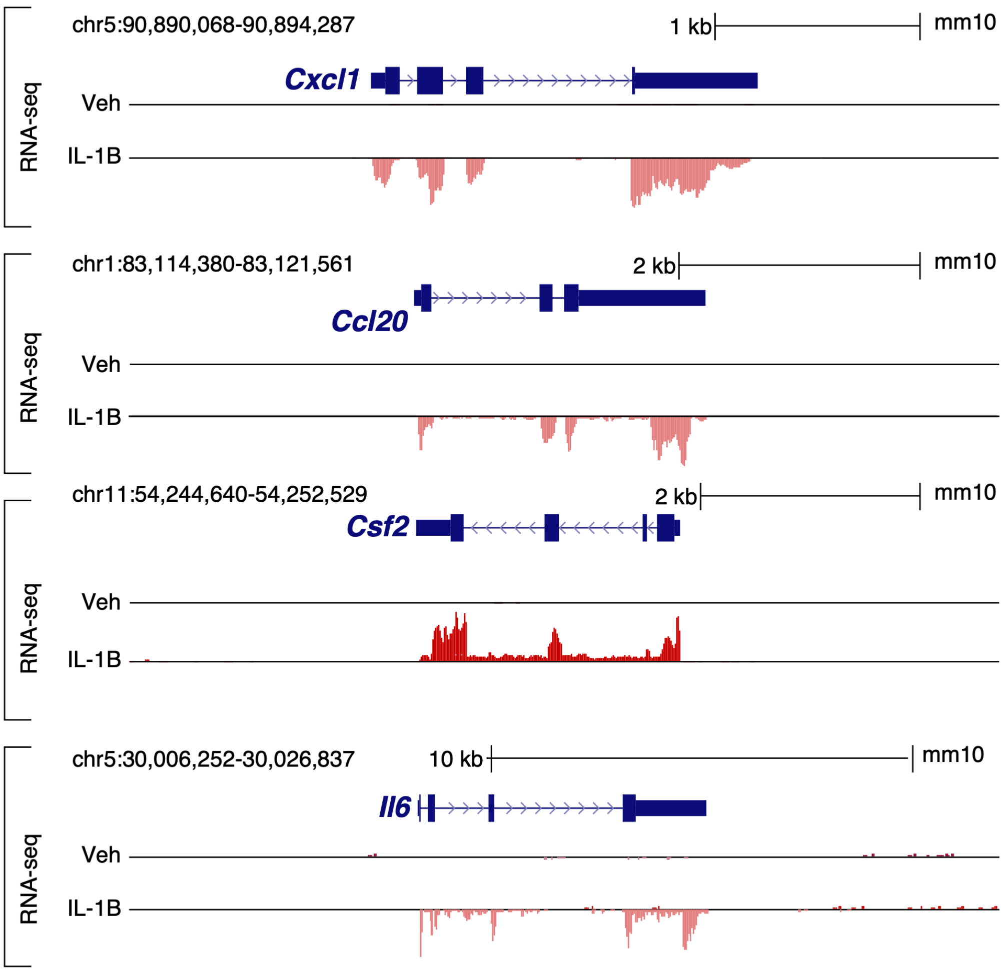


**Figure S1: Representative RNA-seq browser tracks of IL-1B-responsive genes in primary astrocytes.**
Genome browser views showing RNA-seq signal at the *Cxcl1, Ccl20, Csf2,* and *Il6* loci in astrocytes treated with vehicle or IL-1B. These representative examples illustrate the robust transcriptional induction of canonical inflammatory genes following IL-1B stimulation. Gene models and genomic coordinates are shown in *mm10*.

bioRxiv preprint doi: https://doi.org/10.64898/2026.05.03.722406; this version posted May 28, 2026. The copyright holder for this preprint (which was not certified by peer review) is the author/funder, who has granted bioRxiv a license to display the preprint in perpetuity. It is made available under a CC-BY 4.0 International license.

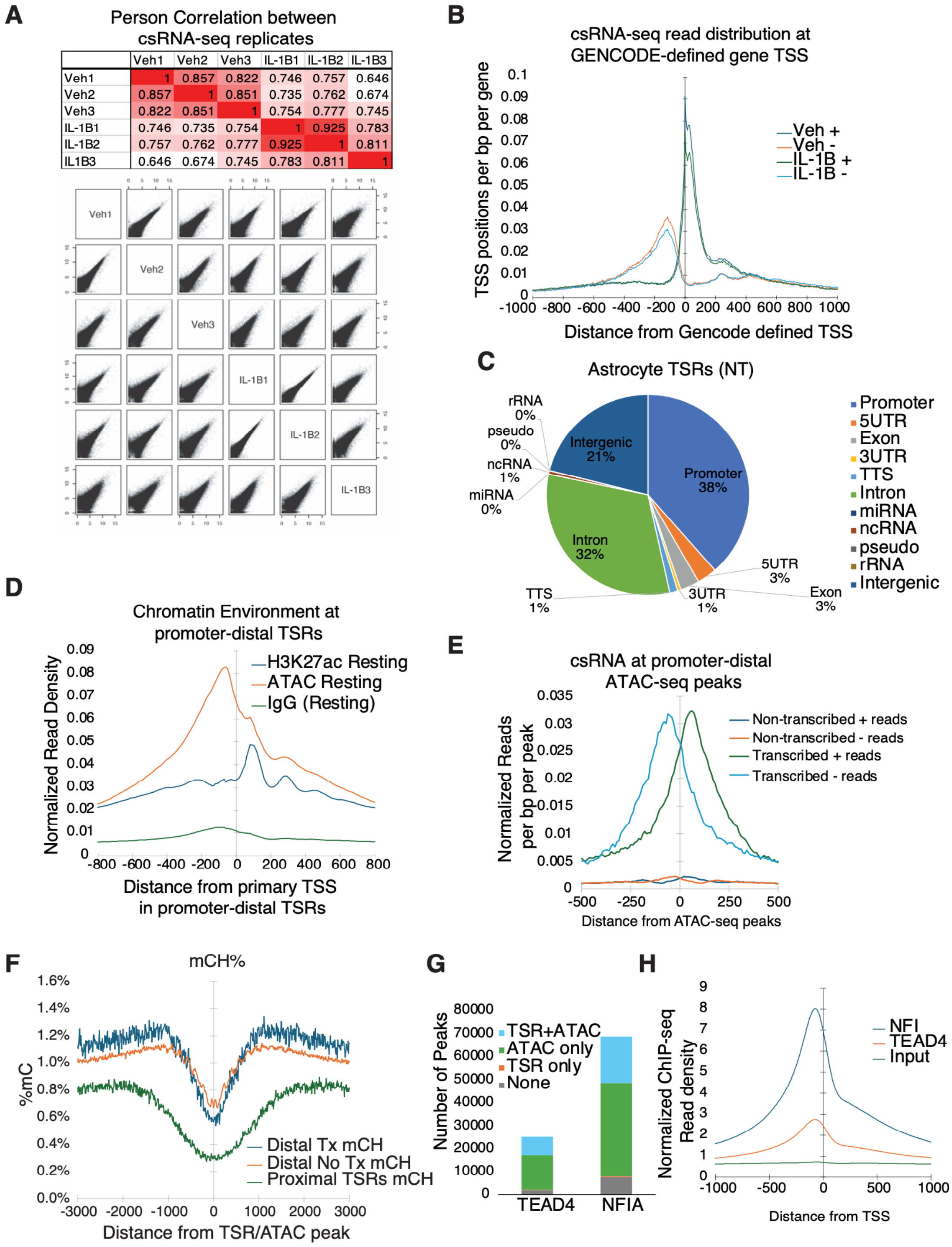


**Figure S2: Features of astrocyte TSRs and associated chromatin environment**
(A) Pairwise correlation analysis of csRNA-seq replicates from untreated and IL-1B-treated astrocytes.
(B) Distribution of strand-specific csRNA-seq reads around GENCODE-annotated TSSs, showing strong enrichment at annotated transcriptional start sites.
(C) Genomic annotation of astrocytes TSRs, partitioned across promoter, intronic, intergenic, and other genomic features.
(D) Average chromatin profiles at promoter-distal TSRs, showing ATAC-seq and H3K27ac enrichment around transcribed regulatory elements.
(E) Average csRNA-seq signal centered on promoter-distal ATAC-seq peaks, comparing transcribed and non-transcribed accessible regions (No Tx: 225,150 peaks, Tx: 14,836 peaks).
(F) Average mCH profiles at promoter-proximal TSRs, promoter-distal transcribed TSRs, and non-transcribed distal accessible regions in astrocytes (data from[50]).
(G) Distribution of NFIA and TEAD4 ChIP-seq peaks found overlapping TSRs and ATAC-seq peaks in astrocytes.
(H) ChIP-seq read density for NFIA and TEAD4 centered on csRNA-seq defined TSRs, showing transcription factor binding immediately upstream of the primary TSS.

bioRxiv preprint doi: https://doi.org/10.64898/2026.05.03.722406; this version posted May 28, 2026. The copyright holder for this preprint (which was not certified by peer review) is the author/funder, who has granted bioRxiv a license to display the preprint in perpetuity. It is made available under a CC-BY 4.0 International license.

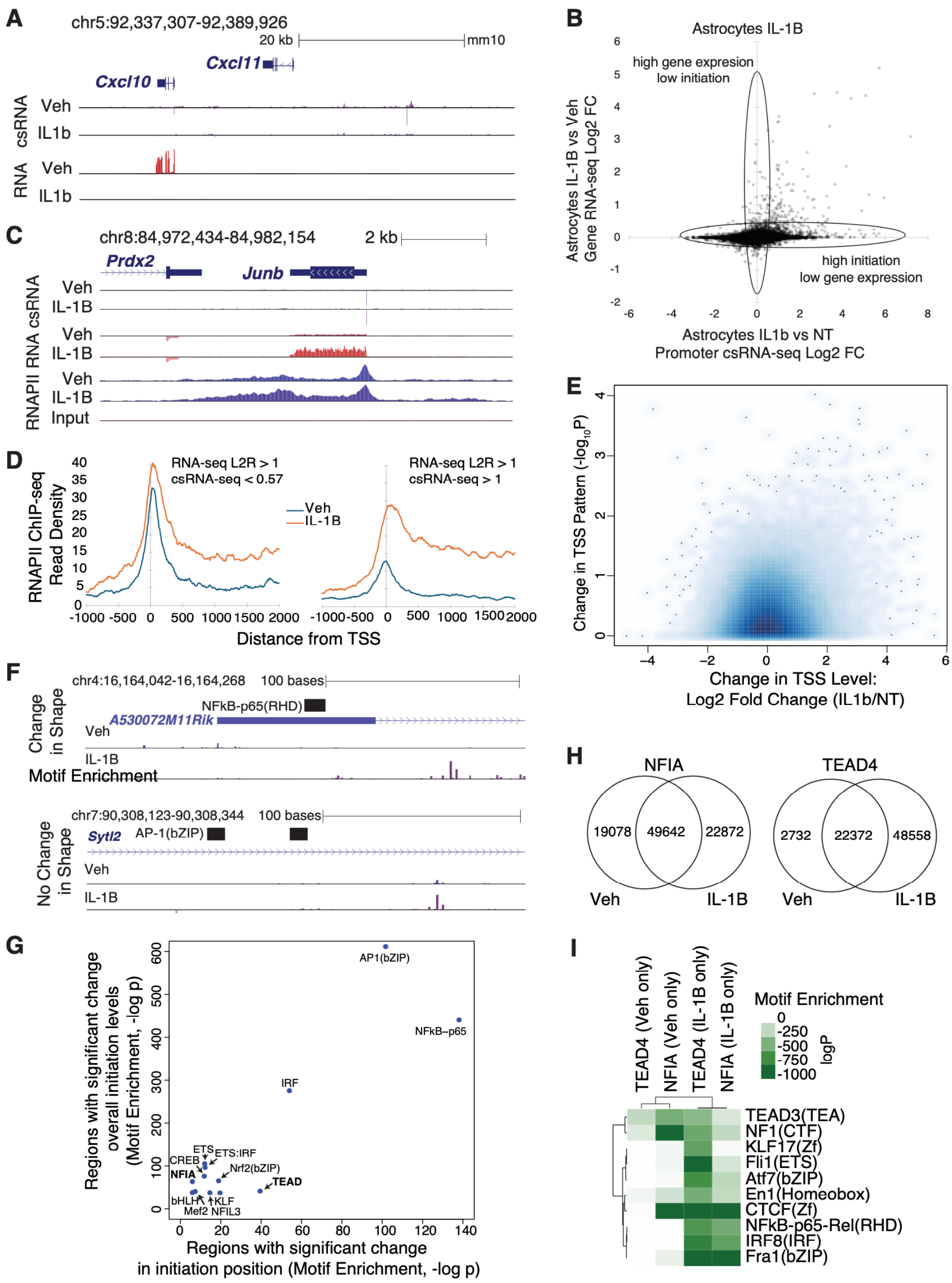

**Figure S3: Distinct effects of IL-1B on transcription initiation level and TSS architecture**

(A) Genome browser example of an IL-1B-induced locus (Cxcl10) in astrocytes, showing increased transcription initiation after stimulation.

(B) Scatter plot comparing IL-1B-induced Log2 csRNA-seq changes at the promoter vs. RNA-seq changes across genes, highlighting genes regulated primarily at initiation (along x-axis) versus those showing stronger changes at the mRNA level (along y-axis).

(C) Genome browser tracks at the Junb locus showing increased gene expression and RNAPII elongation in the gene body with limited change in promoter initiation and RNAPII promoter levels, consistent with regulation being mediated primarily at the level of transcription elongation rather than increased initiation.

(D) RNAPII ChIP-seq levels at the promoters of IL-1B induced genes stratified by genes with minimal versus strong increases in csRNA-seq initiation activity.

(E) Scatter plot comparing changes in overall TSR levels (Log2 Fold change, NT vs. IL-1B) versus their WIP score significance (the -Log10 p-value), identifying TSRs with altered initiation patterns independent of changes in total transcriptional output.

(F) Representative examples of TSRs exhibiting a strong change in initiation pattern (top, WIP score -1.61, Lg10 p-value = 9.54e-05) versus a strong change in overall initiation levels with minimal change in initiation shape(WIP score -0.13, Log10 p-value = 0.84).

(G) Scatter plot of TF motif enrichment in TSRs with significant changes in overall activity versus changes in TSS positions, highlighting differential associations of NF-κB, TEAD, and NFI motifs with these TSRs classes.

(H) Venn diagram showing the overlap of ChIP-seq peaks for NFIA and TEAD4 before and after IL-1B stimulation in astrocytes.

(I) Motif enrichment analysis of condition-specific (either Veh or IL-1B) TEAD4- and NFIA-bound regions, showing IL-1B-specific enrichment for inflammatory TF motifs, including NF-κB, IRF (IRF8), and AP1 (i.e. Fra1).

bioRxiv preprint doi: https://doi.org/10.64898/2026.05.03.722406; this version posted May 28, 2026. The copyright holder for this preprint (which was not certified by peer review) is the author/funder, who has granted bioRxiv a license to display the preprint in perpetuity. It is made available under a CC-BY 4.0 International license.

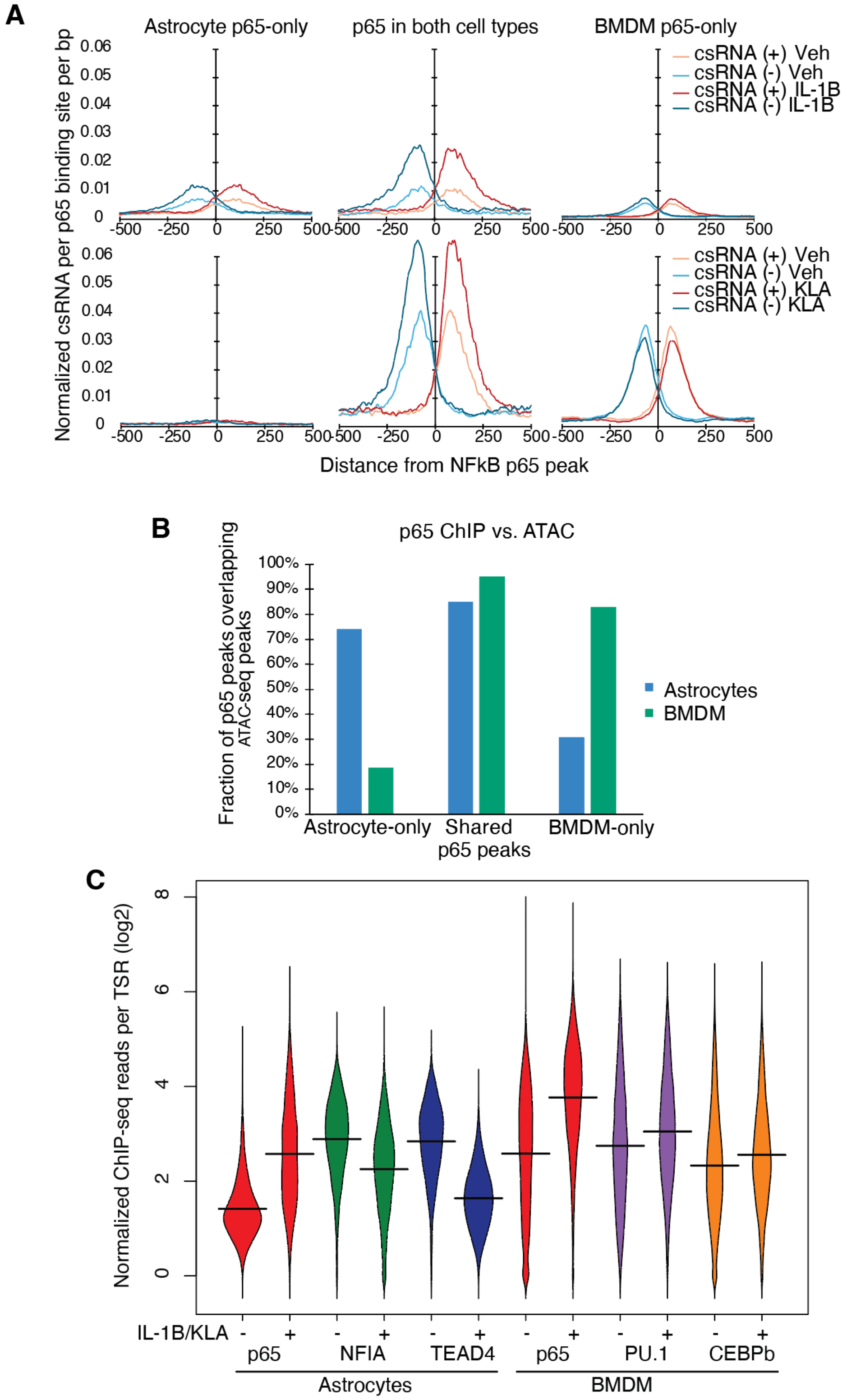


**Figure S4: NF-κB binding is cell-type restricted and linked to pre-existing chromatin and lineage TF occupancy**
(A) Average eRNA signal centered on astrocyte-specific, shared, and BMDM-specific NF-κBp65 peaks, showing cell type-matched induction of regulatory transcription at p65 bound sites.
(B) Fraction of astrocyte-specific, shared, and BMDM-specific p65 peaks overlapping accessible chromatin regions in astrocytes or BMDMs, indicating that NF-κB recruitment occurs preferentially at cell-type-specific open chromatin regions.
(C) Violin plots showing increase in ChIP-seq signal for p65, NFIA, TEAD4 at astrocyte IL-1B-induced TSRs and for p65, PU.1, and CEBPβ in macrophage KLA-induced TSRs.

bioRxiv preprint doi: https://doi.org/10.64898/2026.05.03.722406; this version posted May 28, 2026. The copyright holder for this preprint (which was not certified by peer review) is the author/funder, who has granted bioRxiv a license to display the preprint in perpetuity. It is made available under a CC-BY 4.0 International license.

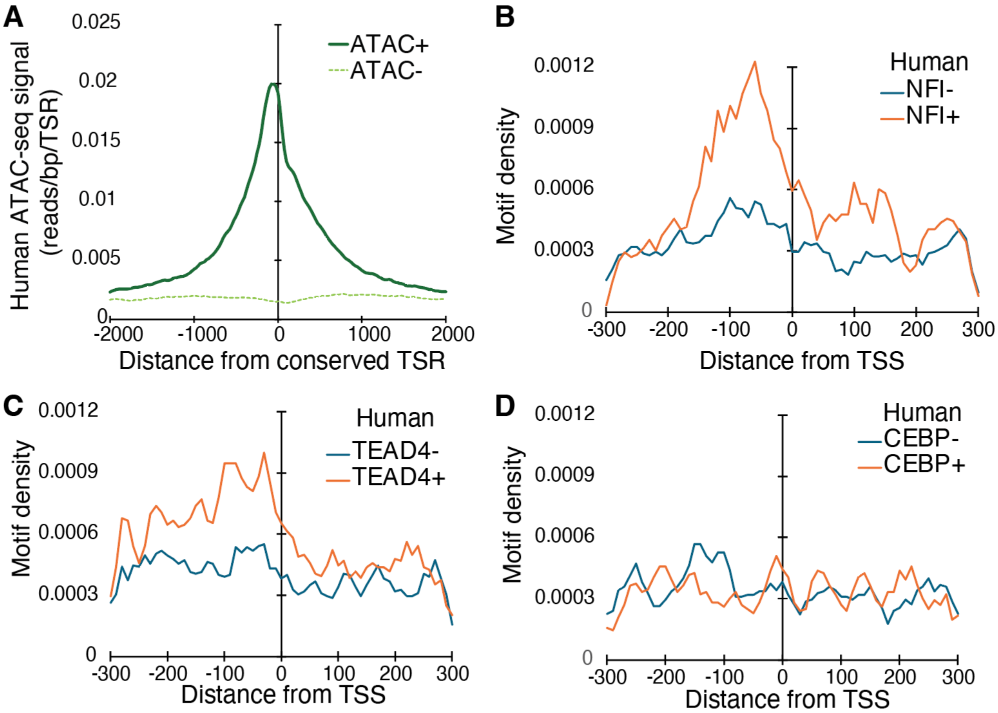


**Figure S5: Human accessibility and motif architecture at conserved astrocyte TSRs**
(A) Distribution of ATAC-seq reads in human astrocytes at conserved mouse TSRs that overlap human ATAC-seq peaks (ATAC+) versus those that do not (ATAC-).
(B) Average human NFI motifs centered on conserved mouse TSRs, stratified by whether the homologous human region is accessible or not.
(C) Average human TEAD4 motifs centered on conserved mouse TSRs, stratified by whether the homologous human region is accessible or not.
(D) Average human CEBP motifs centered on conserved mouse TSRs, stratified by whether the homologous human region is accessible or not.

bioRxiv preprint doi: https://doi.org/10.64898/2026.05.03.722406; this version posted May 28, 2026. The copyright holder for this preprint (which was not certified by peer review) is the author/funder, who has granted bioRxiv a license to display the preprint in perpetuity. It is made available under a CC-BY 4.0 International license.

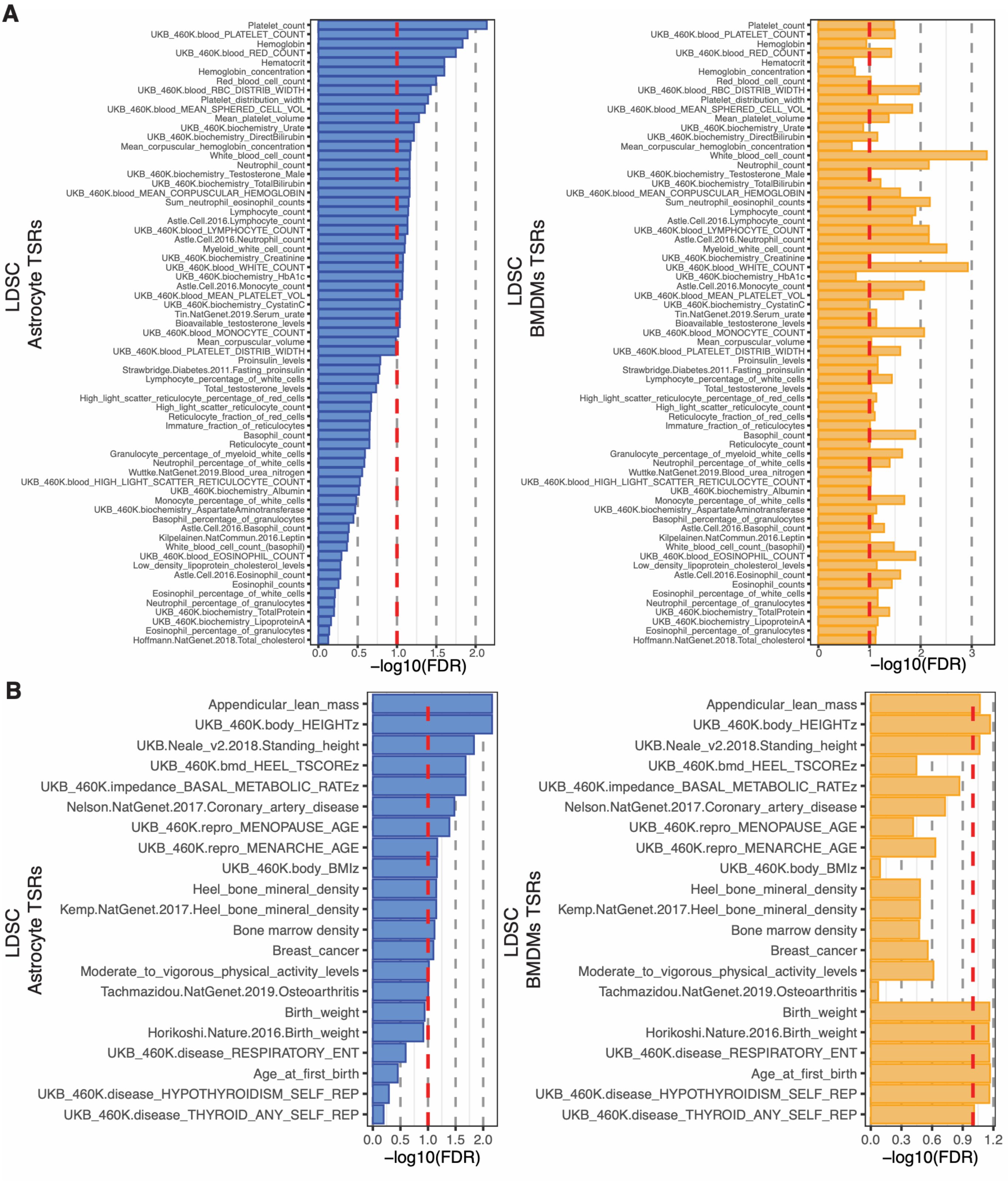


**Figure S6: LDSC-based enrichment of additional GWAS traits**
(A) LDSC regression enrichment for hematological traits across conserved astrocyte TSRs (left) or BMDMs TSRs (right).
(B) LDSC regression enrichment for remaining traits across conserved astrocyte TSRs (left) or BMDMs TSRs (right).

bioRxiv preprint doi: https://doi.org/10.64898/2026.05.03.722406; this version posted May 28, 2026. The copyright holder for this preprint (which was not certified by peer review) is the author/funder, who has granted bioRxiv a license to display the preprint in perpetuity. It is made available under a CC-BY 4.0 International license.

# Supplemental Information

### Supplemental Table 1: Dataset Statistics

List of experiments and read counts for data generated in this study.

### Supplemental Table 2: RNA-seq DEGs

Two worksheets describing genes induced or repressed by IL-1B in primary mouse astrocytes. The final two columns describe genes that are also significantly regulated by KLA in BMDMs.

### Supplemental Table 3: csRNA-seq TSRs

Table describing properties of the 69,435 TSRs identified in mouse astrocytes. Each TSR entry contains information about its position in the mm10 genome, annotation to nearby genomic features and genes, csRNA-seq normalized count information and differential regulation by IL-1B, overlap annotation with ATAC-seq, NF-κB p65 binding, and BMDM KLA regulation, changes in TSS initiation site selection (WIP2 scores), and human conservation information.

### Supplemental Table 4: GWAS enrichment

GWAS enrichment results for overlap between csRNA-seq-defined TSRs and filtered GWAS Catalog loci. Results are reported for each trait-study combination, including total loci, overlapping loci, enrichment statistics, empirical P values, and FDR-adjusted significance. The first sheet includes all tested trait-study combinations, and the second sheet reports the subset of traits related to neurological and neuropsychiatric phenotypes.

### Supplemental Table 5: LDSC

The table reports LDSC enrichment statistics, regression coefficients, standard errors, nominal P values, and multiple-testing-adjusted significance values for each trait and TSR set (astrocytes, BMDMs).
